# An Overview of Current Pretreatment Methods Used to Improve Lipid Extraction from Oleaginous Microorganisms

**DOI:** 10.3390/molecules23071562

**Published:** 2018-06-28

**Authors:** Alok Patel, Fabio Mikes, Leonidas Matsakas

**Affiliations:** Biochemical Process Engineering, Division of Chemical Engineering, Department of Civil, Environmental and Natural Resources Engineering, Luleå University of Technology, 971-87 Luleå, Sweden; alok.kumar.patel@ltu.se (A.P.); fabio.mikes@ltu.se (F.M.)

**Keywords:** oleaginous microorganisms, lipid extraction, pretreatment, cell disruption

## Abstract

Microbial oils, obtained from oleaginous microorganisms are an emerging source of commercially valuable chemicals ranging from pharmaceuticals to the petroleum industry. In petroleum biorefineries, the microbial biomass has become a sustainable source of renewable biofuels. Biodiesel is mainly produced from oils obtained from oleaginous microorganisms involving various upstream and downstream processes, such as cultivation, harvesting, lipid extraction, and transesterification. Among them, lipid extraction is a crucial step for the process and it represents an important bottleneck for the commercial scale production of biodiesel. Lipids are synthesized in the cellular compartment of oleaginous microorganisms in the form of lipid droplets, so it is necessary to disrupt the cells prior to lipid extraction in order to improve the extraction yields. Various mechanical, chemical and physicochemical pretreatment methods are employed to disintegrate the cellular membrane of oleaginous microorganisms. The objective of the present review article is to evaluate the various pretreatment methods for efficient lipid extraction from the oleaginous cellular biomass available to date, as well as to discuss their advantages and disadvantages, including their effect on the lipid yield. The discussed mechanical pretreatment methods are oil expeller, bead milling, ultrasonication, microwave, high-speed and high-pressure homogenizer, laser, autoclaving, pulsed electric field, and non-mechanical methods, such as enzymatic treatment, including various emerging cell disruption techniques.

## 1. Introduction

Over the last decades, the production of biofuels from renewable sources has gained more attention due to critical environmental issues, such as greenhouse gas emission, rapid depletion of fossil fuel supplies, and high energy cost [1]. Microbial oils are found to be a good option to produce biofuels, as many microorganisms, such as microalgae, yeast, bacteria, and fungi have the ability to accumulate oils under special cultivation conditions [2]. Moreover, microbial sources of lipids have many advantages over other sources, including higher lipid productivity in terms of g/L/day, being unaffected by any seasonal climate changes, low labor intensiveness, and easily scale-up [3,4]. The production costs are the major limiting factor to utilize microbial oils for biodiesel production, since the feedstocks to cultivate microorganisms account for 60% to 80% of the overall production cost [5]. To enable the commercial production of microbial lipids, these costs must be reduced by using low-cost feedstocks [5]. Oleaginous microorganisms can utilize various types of organic carbons, regardless of their origin, to accumulate oils in their cytoplasm. They have the specific ability to grow well on inexpensive agricultural waste and industrial by-products [6,7]. Conversion of microbial oil into biodiesel is involving four important upstream and downstream processes, i.e., cultivation, harvesting, lipid extraction, and transesterification [3]. However, the cell disruption process, including lipid extraction from oleaginous microorganisms, is costly and considered as a major bottleneck to produce biodiesel in large scale [8]. The lipids are synthesized intracellularly, which makes downstream processing more problematic for lipid recovery in lab or large-scale [9]. Lipid extraction is usually carried out after the disintegration of cells by pretreatment methods, followed by lipid recovery with organic solvents from the lysed biomass [10]. The cell disruption is an energy-intensive process requiring drying/dewatering of the biomass, which makes the overall process costly [11]. Conventional methods for lipid extraction, such as the Bligh & Dyer as well as the Folch method, involve the use of mixtures of chloroform and methanol which are suitable only for lab-scale [12]. Other problems that are associated with these methods, such as extraction from dry biomass and the use of harmful organic solvents, are also to be taken into account when trying to improve the efficiency of cell disruption [12,13]. Currently, various mechanical, chemical, and enzymatic pretreatment methods are employed to disrupt oleaginous microorganisms on a laboratory scale. These methods include microwave irradiation, ultrasonication, high-speed homogenization, high-pressure homogenization, bead beating, autoclaving, and thermolysis [14]. However, none of these pretreatment methods is effective in higher scale processes [15]. At commercial scale, lipid extraction is usually achieved through a solvent system, where the biomass should be in dry condition, otherwise, the organic solvents cannot establish contact with cells and they remain in the water phase due to their surface charges [16]. However, when considering the expenses involved in drying biomass, the lipid extraction should be done in wet conditions [17]. Disruption of cells by other means also has certain limitations regarding efficiency and lipid yield. For example, extraction of oils from seeds is usually carried out by simple mechanical methods, such as oil press or expeller press, and these methods are also applicable to extract the oils from microalgae. Yet, there is no report for this approach that is used in the extraction of lipids from oleaginous bacteria and yeast [18,19]. For the mentioned techniques, high mechanical pressure is usually applied on the dried biomass to squeeze the oils from cells, but the applied pressure generates excessive heat that can clog the machinery [20]. Although oil press and expeller press are cost-effective methods and they work well with samples of low moisture content, the explored biomass should be moisture-free, otherwise, lipids may pass through the pressed cake [9]. Moreover, the recovery of lipids is not yet sufficient and the drying of biomass again results in high energy and cost demands [21]. Bead beating, another mechanical method, eliminates the drying step, which in turn decreases the overall cost of extraction. In this approach, the wet slurry of biomass spins in a speed rotator that is loaded with fine beads. Since bead beating is only suitable for small amounts of sample, the application on a larger scale is yet again found to be difficult [22]. 

The problems that are associated with conventional methods can be solved with other physical methods, such as microwave irradiation and ultrasonication. Ultrasonication is one of the most extensively used pretreatment method to disrupt the cellular integrity of oleaginous microorganisms. This technique involves the use of mild pressures and temperatures, which makes the method simple, eco-friendly, and less time-consuming. Moreover, it can be operated without using any beads or chemicals. However, one important weak point of this technique is the generation of free radicals after prolonged treatment, which might have a detrimental effect on the quality of the extracted lipids [23]. Besides ultrasonication, microwave treatment is also a commonly used technique to disrupt cells and extract lipids from oily seeds, and it was already applied in the mid-1980s. Microwaves usually affect dielectric or polar particles within the cells, where a high amount of heat is generated during friction of inter- and intra-molecular movement of particles. The vapors generated due to the presence of water in intracellular compartments exert pressure on the cell wall, therefore leading to cell disruption. In this way, microwave irradiation makes membranes porous and plays a significant role in the lipid extraction process. However, this method requires high electricity expenditures that lead to high cost when being applied on a commercial scale [24].

Hence, in order to find a suitable and cost-effective alternative to mechanical methods, many researchers have been involved in replacing them with biological methods. For example, Jin et al. (2012) used recombinant β-1,3-glucomannanase plMAN5C enzymes to disintegrate the cell wall of oleaginous yeast *Rhodosporidium toruloides* Y4 [25].

Recently published literature is focused mainly on the microalgal biorefinery, including pretreatment that is involved in the lipid extraction process. Hence, in the present review article, we are focusing on the various pretreatment techniques employed to improve the lipid extraction process from different types of oleaginous cellular biomass, such as microalgae, yeast, fungi, and bacteria. The discussed pretreatment methods are mechanical methods, such as expression or expeller press, high-pressure homogenization, high-speed homogenization, bead milling, ultrasonication, microwave, autoclave, acid-catalyzed hot-water, laser, and pulsed electric field treatment. Besides these techniques, some other non-mechanical pretreatments are also discussed here. 

## 2. Microbial Cell Wall and Lipid Composition

Cell disruption is the process of breaking indehiscent bacterial cells and cell wall structures of eukaryotic microorganisms, such as yeast, algae, and fungi [3]. The structure of the cell wall varies with the type of microorganism and the given growth conditions. Knowledge of the cell wall structure of a microorganism helps with the selection of a suitable pretreatment method to disrupt its cellular integrity. Disruption of yeast cell walls is more straightforward when compared to bacterial cells due to their larger cell size and a unique cell wall structure [22]. The cell wall of yeasts contains mainly glucans, mannans, and proteins and the overall structure is thicker than in gram-positive bacteria [26]. Pomraning et al. (2015) suggested that oleaginous yeasts start to synthesize lipid droplets in their compartment after 60 to 72 h of growth and that a significant change in the thickness of cell walls can be observed under a transmission electron microscope. Older cells have thicker cell walls than younger ones [26]. Microalgae are also characterized by a thicker cell wall structure made up of complex carbohydrates and glycoproteins. Jiang et al. (2018) observed the cellular ultrastructure of *Chlorella sorokiniana* SDEC-18 under a transmission electron microscope and revealed that the plasma membrane is surrounded by a thick cell wall [27]. 

Oleaginous microorganisms synthesize various kinds of lipid classes in their cellular compartment, which, according to the polarity of their head groups, can be classified as neutral lipids that are acting as energy storage (triacylglycerols, free fatty acids, sterols, sterols esters, waxes, and hydrophobic pigments), and polar lipids that are enabling membrane integrity (phospholipids, glycolipids, polysaccharides, and lipoproteins) [28]. The major proportion of total lipids are triacylglycerides with long-chain fatty acids similar to plant oils, making them comparable to conventional vegetable oil [29]. Triacylglycerols (TAG) are fatty acid triesters of glycerol. There are diverse types of TAG with different properties depending on their fatty acid composition [30].

The occurrence of TAG as storage compounds is widespread among eukaryotic organisms, such as microalgae, yeast, fungi, plants, and animals, whereas the occurrence of TAG in bacteria has only rarely been described [31]. However, there are some interesting species of bacteria, such as *Mycobacterium*, *Streptomyces*, *Rhodococcus*, and *Nocardia*, which can synthesize lipids in quantities of up to 70% of the cellular dry weight. Microbial lipid content and composition varies from one species to another and strongly depends on the cultivation conditions. 

## 3. Conventional Methods for Total Lipid Extraction

### 3.1. Bligh & Dyer Method

The Bligh & Dyer method (1959) is a multistep process for lipid extraction, which is used extensively in the literature (more than 47,700 total citations according to Google Scholar) and its use keeps increasing rapidly [32]. It is considered as the standard method for total lipid extraction. Researchers use either the original protocol or a modified version, according to their convenience. Modifications can be done only at the pretreatment step [9]. Hussain et al. (2014) tried four different methods to extract the lipids from the freeze- and oven-dried oleaginous fungus *Mortierella isabellina* and suggested that the Bligh & Dyer method using methanol:chloroform:water at a ratio of 2:1:0.8 results in the highest lipid yield (41%) from oven-dried fungal biomass [33]. Although the Bligh & Dyer method is widely used, there are some drawbacks. Amongst other limitations, the laborious multistep process is not suitable for large quantities of biomass, and significant amounts of harmful organic solvents are utilized in the process. 

### 3.2. Folch Method 

After the Bligh & Dyer method, the Folch method is the second most used method for lipid extraction from oleaginous microbial biomass [34]. It was initially developed to extract and purify the lipids from brain tissue in a two-step process. In the first step, the lipids are extracted from the homogenized tissue with 2:1 chloroform-methanol (*v*/*v*) and in the second step, the non-lipid substances are removed by phase separation after adding at least five-fold volumes of water to the filtrate from step one [34]. Researchers are using this method with minor modification, e.g., Cheirsilp and Kitcha (2015) used the Folch method for the extraction of lipids from the oleaginous fungus *Aspergillus tubingensis* TSIP9. They applied sonication on the mixture of dried biomass and chloroform:methanol (2:1) for 30 min, followed by filtration [35]. Kumar et al. (2015) used a similar method to extract lipids from homogenized biomass of the oleaginous bacteria *Rhodococcus opacus* with chloroform and methanol (2:1, *v*/*v*), followed by 15–20 min of shaking in an orbital shaker at ambient room temperature [36]. A modification of the Folch method for the extraction and purification of lipids from oleaginous yeast *Cryptococcus terricolus* was described by T. A. Pedersen, where the extracted crude lipids were dissolved in chloroform and methanol (2:1, *v*/*v*) and distilled water, followed by a separation step by centrifugation [37]. Hara and Radin (1978) also tried to extract lipids from the tissues with non-toxic solvents, such as hexane:isopropanol, followed by a washing step with aqueous sodium sulfate to remove non-lipid contaminants from the extract [38]. 

## 4. Pretreatment of Oleaginous Microbial Biomass to Extract Lipids

**Figure 1:**
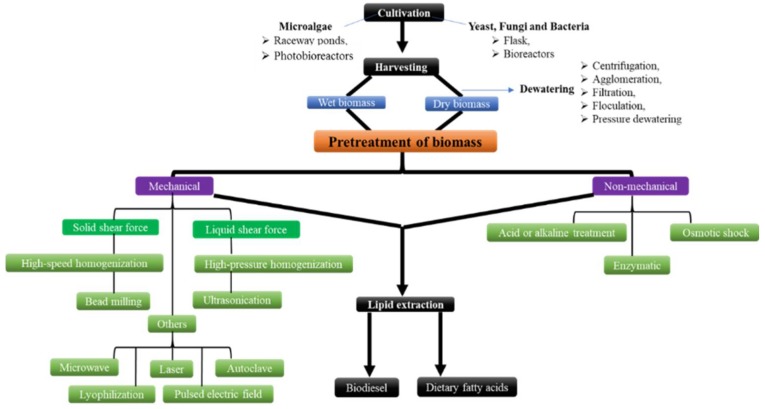
Flowchart showing various pretreatment methods for lipid extraction from oleaginous microbial cells.

The effect of microbial biomass pretreatment on the lipid extraction process has not yet been discussed extensively. The efficiency of lipid extraction varies depending on the pretreatment process that is used to disrupt the cellular integrity as it increases with an increasing degree of cell disruption. However, other parameters, such as residual water content in the case of wet biomass and particulate size in the case of dry biomass, may also affect the pre-treatment process [2,14]. The choice of pretreatment depends on the cellular structure of the microbial biomass. It can be a single-step or a multistep process depending on the physical condition of the biomass (dry or wet). Various pretreatment methods for the disintegration of cellular membranes are currently in use and they can be divided into two main groups of (i) mechanical and (ii) non-mechanical methods (Figure 1). 

Some researchers divide the cellular disintegration into thermal treatment methods and non-thermal treatment methods, whereas others explain the pretreatment methods in combination with lipid extraction and categorize them as mechanical methods, such as oil expeller, ultrasonication, and microwave-assisted extraction or chemical methods, such as Soxhlet extraction, supercritical fluid extraction, and accelerated solvent extraction [20]. All of these pretreatment methods have been extensively utilized for the efficient lipid extraction from various oleaginous microorganisms, such as yeast, microalgae, fungi, and bacteria (Table 1).

### 4.1. Mechanical Pretreatment Methods

Mechanical pretreatment for the disintegration of cellular structure is usually carried out by applying mechanical forces or energy transfer through conventional heat, waves, and electric currents. Mechanical forces can be divided into two forms, i.e., solid-shear forces (e.g., bead mill, high-speed homogenization) and liquid-shear forces (e.g., high-pressure homogenization, micro fluidization). The direct energy transfer to the cells can be achieved by waves (laser, ultrasonication, and microwave treatment), conventional heat (autoclave and water bath), or by applying a pulse electric field [2].

#### 4.1.1. Oil or Expeller Pressing

Expeller pressing is the simplest method to extract oils by mechanical crushing. It has many advantages, like smooth and hands-free operational conditions and low needs for maintenance. The lipids are extracted from the dry biomass by applying mechanical pressure to squeeze out the oils from the broken cells. However, this method is relatively slow and requires large amounts of biomass [65]. The applied pressure must be optimal, otherwise it will result in excessive heat generation and blockage problems due to high pressure [66]. Although this method is usually used to extract oils from seeds, some microalgal lipids have also been extracted with this method. For example, filamentous algae were explored for lipid extraction by using the screw expeller press and almost 75% total lipids were extracted from algae by this method [67]. Depending on the type of biomass, various press configurations, such as screw, expeller, piston, etc., can be used. Some researchers suggest that this method is an expensive and slow process when applied on microbial biomass [24,68]. Johnson and Wen (2009) stated that it is a suitable method for feedstocks, like soybean or canola seeds, where lipids can be extracted from the crushed biomass with solvent, while the extraction process may not be suitable for microalgal cells (both mud-like form and dry powder form of algae), where rigid cell walls hinder the extraction process [69]. Topare et al. (2011) suggested that the solvent extraction by Soxhlet apparatus is effective and extracted more than 98% of the lipids from microalgal cells. But, since it is not a cost-effective method, they tried the expeller press method to extract lipids and this method could recover 75% of the oil from algae [67]. 

#### 4.1.2. Bead Milling 

The history of bead milling goes far back to when it was first applied in the manufacturing of cosmetics to reduce the particle size of paint or lacquer and to grind minerals. After proving its effectiveness in the chemical industry, bead milling was successfully applied for the disruption of microbial cells for the downstream processing of intracellular products [20,70,71,72]. This method has many advantages, such as the need of only single-pass, continuous module of operation, high disruption efficiency, easy biomass loading, mild operating temperature, and applicability to various types of biomass from lab-scale to industrial scale [20,67,71,72]. The operating conditions for the efficient disintegration of cells depend on various factors such as agitator geometry, speed, biomass concentration, slurry flow rate, bead size, bead-to-substrate ratio, etc. Montalescot et al. (2015) reported that disruption of two microalgae, *Nannochloropsis oculata* and *Porphyridium cruentum*, was performed by continuous bead milling where the highest bead filling ratio of >55% *v*/*v* was found to be optimal [73]. The type and size of beads also strongly affect the disintegration of microalgal cells. Doucha and Lívanský (2008) suggested that zirconium oxide (ZrO_2_) beads are more efficient than glass beads for cellular disintegration because of their higher specific density [74]. Postma et al. (2015) reported that kinetic rate constants can be increased by increasing the speed of the agitator as well as the biomass concentration [72]. They investigated the disintegration of *Chlorella vulgaris* by using zirconium oxide (ZrO_2_) beads with a diameter of 1 mm, which gives a lower specific energy consumption, while the agitator speed and biomass loading were 6 m/s and 145 g_DW_/kg [72]. However, similar specific energy consumptions were also achieved by changing the size of beads with similar flow rate and agitator speed [74]. Balasundaram et al. (2012) investigated the optimal balance between shear forces and impact forces that are required for a differential recovery of intracellular products from the cyanobacteria *C. fritschii* (PCC6912) when a custom-made energy efficient ball mill was used for disintegration [70]. Although bead milling is suitable for disintegration of cells, its high energy consumption during operation and its inefficient energy transfer from rotating shaft to individual cells make it an unfavorable method [20]. Oleaginous yeast *Y. lipolytica* IFP29 (ATCC 20460) was disrupted by ultrasound, microwave irradiation, and bead milling and the lipid extraction efficiency was compared to pretreatment with freezing/defrosting, cold-drying, bead milling, and microwave irradiation before the conventional solvent extractions process [75]. It was suggested that bead milling was efficient for lipid extraction from oleaginous yeast biomass while cold-drying under pressure was the best pretreatment method, giving two times more yield when compared to conventional methods [75].

#### 4.1.3. High-Pressure Homogenization

This method is suitable for the stabilization of emulsification processes in cosmetic, pharmaceutical, and food industries, however, it has also been extensively utilized for the microbial cell disruption of microalgae [76], bacteria [77], and yeast [78]. The cell disruption efficiency varies according to the valve seat configurations of the homogenizer [79]. High disruption efficiency is usually achieved through shear forces of highly pressurized fluids on the stationary valve surface and hydrodynamic cavitation from the shear stress induced by pressure drop [80]. High-pressure homogenization has many advantages as it is a simple continuous operating system and can be applied for wet biomass, where the processing fluid is pressurized in intensifiers and passed through a homogenization chamber. The energy is accumulated in the fluid by the pressure and released into the passage through an orifice valve, where the velocity of the fluid increases to up to 200–400 m/s [81,82]. Increased velocity generates mechanical stress, such as shear and elongational forces, turbulence, and cavitation, which are responsible for disruption of cells [83]. Coccaro et al. (2018) suggested that the most efficient disintegration of *Lactococcus lactis* cells was achieved with small orifice valve size, high operating pressure, and low fluid viscosity [77]. In another study, oleaginous microalgae *Nannochloropsis* sp. were disrupted by prior incubation at 37 °C for 15 h before treatment with high-pressure homogenization at 1200 ± 100 bar, followed by lipid extraction with organic solvents [84]. It was a low solvent, low temperature method for efficient lipid extraction from wet concentrated paste where the recovery was reported to be up to 70% *w*/*w* of the total lipids and 86% *w*/*w* of neutral lipids using hexane as solvent [84]. 

#### 4.1.4. High-Speed Shearing Homogenization

High-speed shearing homogenization (HSH) is usually utilized to prepare foams, emulsions, and suspensions [85]. It is a very effective method to disrupt cells, where a slurry of biomass is stirred in a specific device consisting of a stator–rotor assembly with a small gap (100–3000 μm) [86]. The cells are disintegrated due to hydrodynamic cavitation and the shear forces that are caused by stirring at high rpm, which creates high shear rates (20,000–100,000 s^−1^) [20]. High-speed shearing homogenization was used to prepare the extracts of *Agaricus blazei murill* for the extraction of α-glucan with a final carbohydrate content of 96% [86]. Kwak et al. (2018) used a high-shear mixer to disrupt the cells and extract the lipids from the wet biomass of the oleaginous microalgae *Aurantiochytrium* sp. KRS101 [52]. They suggested that the performance of the high-shear mixer was quite similar between wet and dry biomass of microalgae when extracted with different solvents, such as hexane, hexane-isopropanol, and ethanol. The mixtures developed a strong shear stress and cavitation effect when stirred at 15,000 rpm for 10 min, which was enough to extract all the esterifiable lipids from the microalgae [52]. The most important feature of this method is that it can be directly used for high moisture containing samples, thus reducing the water footprint and downstream process costs [86]. However, extensive heat generation and high energy consumption during the operation are the major drawbacks when it comes to scale-up processes [14]. 

#### 4.1.5. Ultrasonication

The ultrasonication method was found to be the most applicable and efficient method for lipid extraction from oleaginous microbial biomass. Cavitation and acoustic streaming are two different phenomena that are created during the application of ultrasound to the cells. Cavitation creates pressure on the cells in the form of microbubbles, leading to the disruption of cell walls and membranes. Ultrasonication for the disruption of microalgal cells has been tested with various types of solvents, such as chloroform-methanol [87,88,89], n-hexane [90,91], diethyl ether [92], and other solvents [87]. 

Three freshwater-isolated microalgal species *Chlorella* sp., *Nostoc* sp., and *Tolypothrix* sp. were disrupted by different methods, such as bead beating, autoclave, microwave, sonication, and 10% sodium chloride solution treatment [93]. Among the tested methods, sonication was found to be the most effective method to disrupt the microalgal cells and the highest lipid content was obtained by disruption of *Chlorella* sp. [93]. The three microalgae species *Phaeodactylum tricornutum*, *Nannochloropsis gaditana,* and *Chaetoceros calcitrans* were tested for lipid extraction with the conventional Bligh & Dyer extraction method (1959) with prior ultrasonication treatment [94]. *Trichosporon oleaginosus* and an oleaginous fungal strain were treated with ultrasonication (520 kHz, 40 W, and 50 Hz, 2800 W) using various solvents, including water, hexane, methanol, and chloroform-methanol (1:1, *v*/*v*), followed by lipid extraction and comparison of the process efficiency to the conventional chloroform-methanol (2:1, *v*/*v*) extraction method [95]. The results suggested that almost all of the lipids (100%) were extracted from *T. oleaginosus* and the SKF-5 strain after a very short incubation (15 min) at a relatively low temperature (25 °C) with chloroform-methanol, followed by pretreatment with ultrasonication at 50 Hz and 2800 W [95]. Mecozzi et al. (2002) performed an experiment for lipid extraction from marine mucilage samples using an ultrasonic cleaning bath at 35 kHz [92]. They used two different solvent systems and suggested that diethyl ether was more suitable than methanol to assist the ultrasonication for lipid extraction. Moreover, the disruption due to the acoustic cavitation phenomena minimized the oxidative damage on the lipids [92]. Wu et al. (2012) investigated the ultrasonication treatment at low frequency (20 kHz) with high intensity (0.0403 W/cm^3^) and found it to be effective for the disruption of *Microcystis aeruginosa* [96]. They suggested that the acoustic cavitation phenomenon at a low ultrasonic frequency is mainly responsible for the damage of cells due to sufficient shear forces being directly applied to cells. But, while the mechanical energy of cavitation is lower at high ultrasonic frequencies, the ultrasonic degradation of water generates free radicals that weaken the cell wall of cyanobacteria [96]. In another study, the lipids were extracted from microalgae *Scenedesmus obliquus* by using sun-, freeze-, and oven-dried biomass. The cells were disrupted by microwave-, sonication-, autoclaving-, and osmotic shock treatment [53]. The results suggested that the lipid yield of dried samples that were subjected to microwave treatment (20.73 ± 4.16%) was higher than for autoclaving and osmotic shock, while the results were comparable with sonication (19.49 ± 3.30%) [53]. Wang et al. (2014) treated two microalgae *Scenedesmus dimorphus* and *Nannochloropsis oculate* with a high frequency focused ultrasound (3.2 MHz, 40 W) and a low frequency non-focused ultrasound (20 kHz, 100 W). The results revealed that high frequency focused ultrasound was a more energy efficient process for maximal cell disintegration [97]. 

#### 4.1.6. Microwave Irradiation

Microwave irradiation is another extensively used method for lipid extraction from oleaginous microorganisms, where electromagnetic waves are applied to the suspension of cells in an organic solvent. During the microwave treatment of polar compounds, the applied alternative current is converted into electromagnetic energy, and finally, in heating energy [98], as the polar compounds align themselves in the direction of the applied electric field and rotate at high speed when the microwave field alters. The process is accelerated when ions are present in the working system [75]. High heat is generated during the frictional movement of polar compounds or ions, not involving conventional radiant heat [99]. Microwave heating consumes almost two to three times less energy than that involves in the conventional heating [100]. Guerra et al. (2014) treated the oleaginous microalgae *Chlorella* sp. with microwaves and enhanced lipid yield was recorded as compared to the conventional Bligh & Dyer method, therefore it further boosts chemical and energy savings. Moreover, lipid extraction using a single-step microwave-assisted extraction was more convenient and effective than the multistep, time consuming traditional Bligh & Dyer method [101]. Furthermore, Lee et al. (2010) stated that the lipid extraction yield was higher for the microwave method when compared to autoclaving, bead beating, ultrasonication, and 10% NaCl solution extraction methods [89]. Microwaves were reported to be a useful tool for the extraction of plant oils and animal fats, and, in addition, their implementation has the advantage of easy scale-up [24]. Teo et al. (2014) performed trials on the extraction of lipids from the marine microalgae *Nannochloropsis* sp. and *Tetraselmis* sp. using four different solvent extraction methods (Hara & Radin, Folch, Chen, and Bligh & Dyer) along with conventional heating and microwave irradiation. The highest lipid yield was obtained when they used the Hara & Radin (8.19%) and the Folch (8.47%) method following the microwave irradiation [102]. Boldor et al. (2010) used microwave treatment for the extraction of oils from Chinese tallow tree in batch and continuous flow mode [24]. They suggested that the application of microwave-assisted solvent extraction to extract the lipids from seeds has many advantages over conventional methods, including short operating time and reduced energy consumption [24]. The cells of three freshwater-isolated microalgal species, *Botryococcus* sp., *Chlorella vulgaris* and *Scenedesmus* sp., were disrupted by different methods, such as bead beating, autoclave, microwave, sonication, and 10% NaCl treatment [89]. Among all of the tested methods, the microwave oven was found to be the simplest, easiest, and most effective method to disrupt the microalgal cells. The highest lipid content was obtained by the disruption of *Botryococcus* sp. [89]. Although microwave treatment is a suitable technique to extract lipids in a short amount of time, it has some drawbacks. Its use is limited to polar solvents and the method is unsuitable for volatile compounds. Moreover, the formation of free radicals and the increased temperature make microwave treatment less favorable [103]. 

#### 4.1.7. Autoclaving

Autoclaving is usually utilized for the sterilization of laboratory equipment and media prior to the growth of microorganisms. Various microalgal species, such as *Haematococcus pluvialis* [104], *Botryococcus* sp., *C. vulgaris*, and *Scenedesmus* sp. [89] were disrupted by autoclaving at 121 °C and 1.5 MPa for 5 or 30 min [89,104]. Rakesh et al. (2015) treated four oleaginous microalgae, *Chlorococcum* sp. MCC30, *Botryococcus* sp. MCC31, *Botryococcus* sp. MCC32, and *Chlorella sorokiniana* MICG5, with various methods, such as autoclaving, microwave irradiation, osmotic shock treatment, and pasteurization, and reported that the highest amount of nutraceutically important unsaturated fatty acids was obtained when *Botryococcus* sp. was treated with autoclaving [105]. Similarly, Florentino de Souza Silva et al. (2014) suggested that autoclaving is a more efficient technique than ultrasonication but not as efficient as microwaving and electroflotation by alternating current (EFAC), when mixed cultures of microalgae were treated with different methods [106]. 

#### 4.1.8. Pulsed Electric Field

Pulsed electric field (PEF) treatment works based on electroporation phenomena, including electromechanical compression and electric field-induced tension, where an external electric field is used to induce the critical electrical potential across the cell membrane [107,108]. The increase in membrane porosity is directly proportional to the strength of the applied electric field and pulses and the pore formation in the membrane can be reversible or irreversible, depending on the size and number of pores in comparison to the total surface area of the membrane or cell wall [107]. Eing et al. (2013) treated the oleaginous microalgae *Auxenochlorella protothecoides* with a PEF at 35 kV/cm and the pulse duration was set to 1 µs. They suggested that the lipid yield after PEF treatment and extraction with ethanol was four times higher than it was for untreated cells [109]. Similarly, the oleaginous microalga *Synechocystis* PCC 6803 was treated with a pulsed electric field (intensity > 35 kWh/m^3^) and isopropanol as solvent [110]. 

In another study, oleaginous microalgae *Ankistrodesmus falcatus* wet biomass was treated with PEF using the green solvent ethyl acetate and the results demonstrated that the lipid yield was 83–88% higher when compared to the untreated cells [111]. 

#### 4.1.9. Laser

Laser treatment is a well-known technique to disintegrate the cellular membrane without damaging the compartments of the cell factory or other interior compounds. Most importantly, laser treatment is free of the use of any organic solvent, fast, and requires no laborious effort [112,113]. Previously, researchers have studied the efficiency and mechanism of this method of cell lysis in static mode for different microorganisms, like *Escherichia coli*, *Saccharomyces cerevisiae*, and microalgae at various wavelengths and energy inputs. In a study, oleaginous microalgae *N. oculata* cells were disrupted by using various pretreatment methods, such as microwave, water bath, blender, ultrasonic, and laser treatment, and it was revealed that the highest disruption efficiency was achieved with laser treatment (96.53%), followed by microwave treatment (94.92%) [114]. However, the number and scope of these studies are limited and further investigations, especially in the continuous system, are required in order to examine the potential applications of this cell disruption method. 

#### 4.1.10. Acid-Catalyzed Hot-Water

Hot water treatment is a well-known technique to disintegrate the crystalline nature of cellulosic biomass [115]. Hot water requires high pressure at an elevated temperature in order to remain in liquid form. It is to be noted that this pretreatment under acidic conditions is applicable for the extraction of lipids from biomass in wet condition ensuring the cost-effectiveness since no extra energy input is required for dewatering processes. Lipids with high free fatty acid content were extracted from *C. vulgaris* by using acid-catalyzed hot-water treatment and the anionic surfactant sodium dodecyl benzene sulfonate (SDBS) [116]. The lipid extraction yield was 266.0 mg/g of cell weight from a total fatty acid content of 296.0 mg/g of cell weight, when the concentration of sulfuric acid and SDBS were 2.0% and 0.2%, respectively [116]. *C. vulgaris* cells were disrupted for efficient lipid extraction using acid-catalyzed hot-water treatment [117]. The lipid extraction yield was 337.4 mg/g of cell weight from a total fatty acid content of 381.6 mg/g of cell weight, given a 1% sulphuric acid concentration and heating at 120 °C for 60 min, when compared to 83.2 mg/g of cell weight lipid yield with no heating and no catalyst [117]. This method is also suitable to extract lipids rich in docosahexaenoic acid (DHA) from *Aurantiochytrium* sp. [118]. During acid-catalyzed hot-water treatment, cells are disrupted along with the degradation of other cellular components, leading to excess acid loading and devaluation of co-products. 

### 4.2. Non-Mechanical Pretreatment Methods

Conventional mechanical techniques have several drawbacks, including insufficient extraction yields, the use of toxic solvents, and long processing time. Hence, there is a need for rapid, less energy intensive methods for the lipid extraction from wet biomass. Non-mechanical disruption methods, such as enzymatic and chemical cell lysis, are mainly used in lab-scale processes for bioanalytical purposes. The energy consumption of mechanical methods is always higher when compared to non-mechanical methods. Lee et al. (2012) compared the energy consumed by lipid extraction from microalgae with the energy levels that are required for other methods [119]. The advantages and disadvantages along with process parameters of various cell disruption methods are summarized and compared in Table 2. 

#### 4.2.1. Enzymatic Pretreatment

The extraction of lipids using enzymatic pretreatment completely depends on the cell wall characteristics of the subjected oleaginous microorganism [130]. This technique includes various cell wall degrading enzymes, such as xylanase, cellulase, amylase, papain, pectinase, and hemicellulase [131]. Enzymatic pretreatment is a well-known technique in the vegetable oil industry to degrade the structural polysaccharides of the cell wall of oily seeds [134,135]. It constitutes a favourable cell disintegration method due to its specificity and mild operating temperature, as well as its low time and energy requirements. Furthermore, the method is devoid of harmful solvents and harsh physical conditions, such as shear forces [132]. It has been reported that enzymatic pretreatment is suitable for extracting lipids from the oleaginous yeast. For example, the oleaginous yeast *Rhodosporidium toruloides* was treated with the recombinant β-1,3-glucomannanase plMAN5C, and almost 96.6% of the total lipid content was extracted directly from the culture with ethyl acetate at room temperature and atmospheric pressure without dewatering [25]. Moreover, this method has also been applied to extract lipids from oleaginous microalgae. The microalga *C. vulgaris* was treated with cellulases for 72 h and the hydrolysis efficiency of the cell wall carbohydrates was 85.3%. After enzymatic hydrolysis, the lipid extraction efficiency by solvent extraction was higher than without hydrolysis [136]. Bonturi et al. (2015) extracted lipids from intact and pretreated cells of oleaginous yeasts *R. toruloides* and *L. starkeyi* using various methods such as Folch, Pedersen, hexane, and Bligh & Dyer methods involving acid and enzyme pretreatment. They suggested that enzymatic pretreatment is not an efficient technique for *L. starkeyi* due to the sulfide bonds in its cell wall, which increase the strength and rigidity of its organelles [47]. 

In another study, the oleaginous microalga *Scenedesmus* sp. was treated with various enzymes, such as cellulase, xylanase, and pectinase under varying conditions, including enzyme concentration, temperature, pH, and incubation time [50]. The results demonstrated that the combination of cellulase, xylanase, and pectinase for 190 min improved the lipid extraction yields by 96.4% when compared to the untreated microalga [50]. Another oleaginous marine microalga, *Nannochloropsis* sp., was treated with cellulase and mannanase and the results revealed the improvement of lipid extraction yields from 40.8% to over 73% [137]. Treatment of the same microalga with similar enzymes under different conditions significantly improved the recovery of lipids from *Nannochloropsis* sp. biomass [133]. The enzymatic hydrolysis of *Chloroccum* sp. by using cellulase obtained from *Trichoderma reesei*, ATCC 26921, was an effective method to enhance the saccharification process of microalgal biomass for bioethanol production [138]. Hence, enzymatic treatment can improve the lipid extraction from various oleaginous microorganisms and scaling up the process is relatively easy. However, long processing times and high capital costs hinder the scale-up of enzymatic pretreatment for lipid extraction in the biorefineries [138]. 

#### 4.2.2. Other Emerging Methods for the Extraction of Lipids from Oleaginous Microorganisms

Researchers have used a limited number of other pretreatment methods, including chemical treatments to disrupt the microbial cells. Supercritical fluid extraction is an extensively used method to extract lipids from oleaginous microbial biomass [9,127,130,139,140,141,142]. Bai et al. (2014) used free nitrous acid (FNA) as an effective and low cost pretreatment method to extract lipids from microalgae [143]. Boyd et al. (2012) used switchable hydrophilicity solvents, such as *N*,*N*-dimethylcyclohexylamine for the lipid extraction from freeze-dried samples of *Botryococcus braunii* microalgae [144]. Two microalgae species, *N. oculata* and *Dunaliella salina*, were treated with a novel simultaneous distillation and extraction process (SDEP) for lipid extraction under wet conditions by using d-limonene, a-pinene and p-cymene as solvents [145]. Du et al. (2013) tried to use switchable solvents such as secondary amines for the extraction of lipids from wet and non-broken algae [146]. Kim et al. (2012) used a mixture of ionic liquid [Bmim][CF_3_SO_3_] and methanol for the extraction of lipids from *C. vulgaris* [147]. Lee et al. (2013) treated *Chlorella* sp. biomass with organic nanoclays, such as Mg–APTES clay, Al–APTES clay, Ca–APTES clay, and Mg–N3 clay [148]. In other reports, researchers used H_2_O_2_ with or without FeSO_4_ to disrupt the cell walls of *C. vulgaris* [149]. Jo et al. (2014) reported a quick method for dimethyl carbonate-mediated lipid extraction from *Chlorella* sp. [150]. Hydrothermal liquefaction (HTL) is the most emerging technique to convert high moisture algal biomass to crude biooil, producing more lipids per mass of microalgae than the other extraction method [151]. This technique is more advantageous than the traditional thermochemical conversion processes, such as pyrolysis, where high energy is required for drying the biomass [152]. In this process, biomolecules are decomposed in hot compressed water via the combined action of elevated temperature, elevated pressure, and hydrolytic attack. Reaction temperature and catalysts are two important factors in hydrothermal liquefaction that decide the fractionation of water-soluble and water-insoluble biocrude from algae [153]. Sheehan and Savage (2017) developed a kinetic model for hydrothermal liquefaction to predict the biocrude yield from protein, lipid, and carbohydrate rich microalgae, and suggested that feedstocks containing more proteins or lipids give higher biocrude yields than those that are abundant in carbohydrates [154]. For example, Hietala et al. (2016) performed isothermal and non- isothermal hydrothermal liquefaction with microalgae *Nannochloropsis* sp. and predicted that up to 46% *w*/*w* biocrude yields are achievable with short holding time of 1 min at 400 °C [155]. 

## 5. Conclusions and Recommendations

This review article summarizes the various techniques that are available for total lipid extraction from the biomass of various oleaginous microorganisms using different mechanical and non-mechanical pretreatment methods. Since all mechanical pretreatment methods are followed by the solvent extraction of lipids, overall costs of downstream processing can be increased. Some researcher use green solvents instead of potentially harmful chloroform and methanol to make the extraction process more feasible. Supercritical fluid extraction techniques appear to be a good option to avoid toxic solvents, but the initial capital cost for equipment is high. Another alternative, involving the use of solvent-free extractions, is offering an environment-friendly and cost-effective option on a laboratory scale. However, more research is required in order to scale up the process. Drying of biomass prior to pretreatment is another energy-intensive step, so the target should be to extract lipids from wet cellular biomass to avoid extra cost for drying. Several pretreatment methods allow for the extraction process in wet conditions. For example, hydrothermal liquefaction is an emerging method in which wet microalgae are converted into crude biooil and this technique is also suitable for scale-up of process. However, using only one single pretreatment method may not be sufficient to reach a maximal lipid extraction yield from lysed biomass, hence it may be advantageous to apply multiple different pretreatment methods on both lab- and large-scale. For example, if ultrasonication and microwave irradiation are used in combination for the cell disruption under wet conditions, four different physical phenomena would work together to easily break the cells and release the lipids to the external environment. During ultrasonication, the transmission of sonic waves causes cavitation where microbubbles form during the rarefaction phase of the sound wave and collapse during the compression phase. Disintegrated microbubbles release shock waves in the form of mechanical energy, which causes irreparable shearing in the cell wall of oleaginous microorganisms. After the ultrasonication step, microwave treatment causes the alignment of polar compounds in the cellular compartments in the direction of the applied electric field followed high-speed rotation when the microwave field alters. With this method combination, both the duration of the process as well as the solvent consumption could be decreased. 

## Figures and Tables

**Table 1:** Comparison of various pretreatment methods for lipid extraction from oleaginous microbial cells.

Oleaginous Micro-Organism	Lipid Extraction Method	Pretreatment of Cells	Lipid Content (%, *w*/*w*)	References
**Oleaginous yeasts**				
*Rhodosporidium kratochvilovae* HIMPA1	Bligh & Dyer method	Ultrasonication at 40 Hz for 5 min	59.7	[39]
	Organic-solvent n-hexane	Acid-catalyzed hot-water treatment	61.9	
	Organic-solvent n-hexane	Microwave irradiation	67.4	
	Organic-solvent n-hexane	Rapid ultrasonication-microwave treatment	70.1	
*Cryptococcus curvatus* (DSM 70022)	Solvent extraction (chloroform-methanol; 2:1, *v*/*v*)	Dried biomass, Acid-catalyzed hot-water treatment. (2 mL of 3 M HCl and then digested at 60 °C for 2 h), Sonication for 30 s at 30 kHz	NA	[40]
			46	
*Rhodotorula glutinis* (DSM 10134)				
			48.9	
*Yarrowia lipolytica* (DSM 8218)				
*C. curvatus* MUCL 29819	Solvent extraction (chloroform-methanol; 1:1, *v*/*v*)	Dried yeast cells, Bead milling (glass beads, diameter 0.5 mm)	30.3	[41]
*Sporidiobolus pararoseus* KM281507	Bligh & Dyer method	Vortexed with glass beads, sonicated at 70 Hz for 30 min	30.7	[42]
*S. pararoseus* KX709872	Bligh & Dyer method	Vortexed with glass beads for 30 min in the presence of 100 ppm ascorbic acid and sonicated for 30 min in ultrasonication bath	56.6	[43]
*Naganishia liquefaciens* NITTS2	Solvent extraction (chloroform-methanol; 1:1, *v*/*v*)	Ultrasonication at 20 kHz for 20 min at 40 °C	55.8	[44]
*C. curvatus* MTCC 2698	Bligh & Dyer method	Sonication at 40 kHz for 2 min	28.3	[45]
*Cryptococcus vishniaccii*	Bligh & Dyer method	Sonication at 20 kHz for 5 min	52.3	[46]
*Rhodosporidium toruloides* and *Lipomyces starkeyi*	Bligh & Dyer method	Acid (2 mol/L of HCl)	25 and 34	[47]
		None	23 and 7	
	Folch method	Acid (2 mol/L of HCl)	34 and 48	
		Enzymatic	31 and 37	
		None	42 and 47	
**Oleaginous microalgae**				
*Schizochytrium* sp. ATCC20888	Soxhlet extraction	Enzymatic lysis with alkaline protease	63	[48]
*Chlorella vulgaris*/*Cyanobacteria leptolyngbya*	Solvent extraction with hexane or chloroform-methanol (1:1, *v*/*v*)	Sonicated in an ultrasonic reactor with a clamp-on transducer	16	[48]
*Phaeodactylum tricornutum*	Solvent extraction (chloroform-methanol; 1:1, *v*/*v*)	Lyophilization	47	[49]
*Scenedesmus* sp.	Solvent extraction (chloroform methanol; 1:1, *v*/*v*)	Enzymatic treatment with cellulase, xylanase and pectinase	86.4 (lipid recovery)	[50]
*Tetraselmis* sp. KCTC12429BP	Solvent extraction with mixture of hexane and polar solvents (ethanol, isopropanol, methanol, tetrahydrofuran, acetone, acetonitrile)	Lyophilization	5.5 with Chloroform-methanol, 5.2 with hexane-methanol	[51]
*Aurantiochytrium* sp. KRS101	Solvent extraction with chloroform, chloroform-methanol (2:1, *v*/*v*), hexane, hexane-isopropanol (3:2, *v*/*v*), methanol and ethanol	High shear mixer (HSM)	High non-esterifiable lipids with chloroform-methanol and esterifiable lipids with chloroform	[52]
*Scenedesmus obliquus*	Solvent (chloroform-methanol; 2:1, *v*/*v*)	Drying of biomass by sun, freeze, and oven followed by microwave, sonication, autoclaving, osmotic shock (10% NaCl)	Highest lipid content of 25.4% was obtained after freeze-drying followed by microwave digestion	[53]
*Scendesmus dimorphus*	Solvent extraction with ethanol (6 mL/g dry algae), Fractionation with (ethanol: hexane: water; 1:1:1, *v*/*v*/*v*)	Extraction autoclave equipped with condenser, mechanical stirring and thermocouple	Oil extraction by fractional method gave neutral lipid (97) with polar lipids (2)	[54]
**Oleaginous fungus**				
*Mucor circinelloides* URM 4182	Solvent extraction With ethanol (96%)	Microwave irradiation at 60 °C for 30 min	31.2	[55]
*Cunninghamella echinulata*	Soxhlet extraction with diethyl ether anhydrous at 50 °C	Dried biomass ground in a laboratory blender	22.2	[56]
*M. circinelloides* VI04473 and *Mortierella alpina* UBOCC-A-112046	Folch method, Bligh & Dyer method	Acid hydrolysis with 2 mL 3 N HCl (incubation of the sample at 80 °C for 1 h), bead beating and homogenization (4.0 m/s for 60 s)	NA	[57]
*M. circinelloides* VI 04473, *Umbelopsis isabellina* UBOCC-A-101350 and *Penicillium glabrum* FRR 419	Lewis extraction	Freeze-dried, biomass, glass beads in high-speed benchtop homogenizer at 6.5 m/s, for 1 min cycle length and 6 cycles	Highest lipid content was obtained from *U. isabellina* at 30 °C	[58]
*Alternaria alternata*, *Cladosporium cladosporioides*, *Epicoccum nigrum*, *Fusarium oxysporum*, *Aspergillus parasiticus* and *Emericella nidulans* var. lata	Folch method	NA	Highest lipid content (40.8) from *A. alternata*	[59]
*Aspergillus tubingensis* TSIP9	Folch method	Slurry of biomass and chloroform-methanol sonicated for 30 min	39.5 mg per gram dry substrate (gds)	[35]
**Oleaginous bacteria**				
*Acinetobacter baylyi* ADP1	Bligh & Dyer method	Freeze-dried cells, vortexed	1.6 with wild strain, 12.4 with genetically modified strain	[60]
*Rhodococcus opacus*	Folch method	Homogenized with chloroform-methanol (2:1, *v*/*v*), followed by shaking	71 with synthetic medium	[36]
*Bacillus subtilis* HB1310	Bligh & Dyer method	4 M HCl, incubation at 80 °C for 1 h	39.8	[61]
*Rhodopseudomonas palustris* (strain 42OL)	Solvent extraction methanol-chloroform (1:2, *v*/*v*)	Grinding of freeze-dried bacterial cells in a mortar with sand	22 to 39	[62]
*Bacillus* sp. V10	Bligh & Dyer method	Freeze-drying of the cells	7.4	[63]
*R. opacus*	Folch method	Homogenized with chloroform-methanol (2:1 *v*/*v*), followed by shaking	65.8	[64]

NA, not available.

**Table 2:** Summary and comparison of various mechanical and non-mechanical pretreatment methods for cellular degradation.

Pretreatment Methods	Mode of Action	Energy Consumption	Scale-Up Possibility	Advantages	Disadvantages	References
Ultrasonication	Cavitation, acoustic streaming and liquid shear stress	Medium/low	Yes/no	Less processing time, lower solvent consumption, greater penetration of solvent into cellular compartment	High power consumption, difficult to scale up	[23,91,92,96,97,120,121]
Oil/expeller press	Mechanical compaction and shear forces	High	Yes	Easy process, no solvent	Large amount of sample required, slow process, unsuitable for samples with high moisture content	[67,122]
High-speed homogenization	Cavitation and shear forces	High/medium	Yes	Simple process, effective, short contact time	High energy consumption, increased temperature during operation	[20,85,86]
High-pressure homogenization	Cavitation and shear forces	High/medium	Yes	Solvent-free, simple process, effective, short contact time	High maintenance cost, less efficient with filamentous microorganisms, no residual effect	[22,123,124,125]
Bead milling	Mechanical compaction and shear forces	High/medium	Yes	Solvent-free, suitable for samples with high moisture content	Low efficiency with rigid cells, depending on various parameters such as bead size and agitation, no residual effect	[19,72,73,75,103,124,126,127,128]
Microwave irradiation	Temperature increase, molecular energy increase	High/medium	Yes/no	Eco-friendly, reduced processing time and solvent consumption	Filtration or centrifugation is necessary to remove the solid residue, unsuitable for non-polar or volatile compounds	[78,95,106,107,128,129,130]
Pulsed electric field treatment	Pore formation due to electric waves	High	Yes/no	Relatively simple, high energetic efficiency, relatively fast	High maintenance costs, high temperature, dependence on medium composition, decomposition of fragile compounds	[20,56,107,108,109,110,111]
Enzymatic treatment	Specific enzyme-substrate interaction	Low	Yes	Simple, high energetic efficiency	Long processing time and high capital cost	[22,25,71,123,125,130,131,132,133]
